# Impact of Obesity on Pregnancy Outcome in Different Ethnic Groups: Calculating Population Attributable Fractions

**DOI:** 10.1371/journal.pone.0053749

**Published:** 2013-01-14

**Authors:** Eugene Oteng-Ntim, Julia Kopeika, Paul Seed, Symon Wandiembe, Pat Doyle

**Affiliations:** 1 Department of Women’s Health, Guy’s and St Thomas’ National Health Service (NHS) Foundation Trust (King’s Health Partners), St Thomas’ Hospital, London, United Kingdom; 2 Faculty of Epidemiology and Population Health, London School of Hygiene and Tropical Medicine, London, United Kingdom; INRA, France

## Abstract

**Objectives:**

To quantify the proportion of adverse pregnancy outcome attributable to maternal obesity.

**Design:**

Cross sectional analysis of routine obstetric dataset.

**Setting:**

Guy’s and St Thomas’s NHS Foundation Trust (GSTFT).

**Population:**

23,668 women who had singleton deliveries at GSTFT between 2004 and 2008.

**Methods:**

Logistic regression was used to estimate the association between BMI and outcome in different ethnic groups. Adjusted odds ratios, and the proportions of obese women, were used to calculate population attributable risk fractions (PAFs).

**Main Outcome Measures:**

(i) Maternal outcomes: diabetes, type of delivery, post-partum haemorrhage, and preterm delivery. (ii) Perinatal outcomes: macrosomia, low birth weight, admission to neonatal intensive care/special care baby unit, and perinatal death.

**Results:**

The prevalence of maternal obesity was 14%. Increasing BMI was independently associated with increasing risk of adverse obstetric and neonatal outcome. At the individual level, the effect of obesity on diabetes was highest in Asian women compared to white women (p for interaction = 0.03). Calculation of population attributable risk fractions demonstrated that one third of diabetes cases and one in six Caesarean sections could be avoided in this population if all obese women were of normal BMI. At the population level, the contribution of obesity to diabetes was highest for Black women (42%), and lowest for oriental women (8%). Seven percent of neonatal macrosomia in all the population, and 13% in Black mothers, were attributable to obesity.

**Conclusions:**

Preventing obesity prior to pregnancy will substantially reduce the burden of obstetric and neonatal morbidity in this population. This reduction will be higher in Black women.

## Introduction

Over half of the women of childbearing age in most developed countries are either overweight (BMI 25–29.9 kg/m^2^) or obese (≥30 kg/m^2^) [1]. It has been estimated that at the start of pregnancy around one in six women in England are obese [2]. Women who are obese pre-pregnancy face an increased risk of adverse obstetric outcomes [3]–[4]. These risks include gestational diabetes [5], pre-eclampsia [6], thromboembolism [7], increasing caesarean section [8] and perinatal morbidity and mortality [9]–[10]. However, most published research has been done in predominantly White populations with less than 10% black and ethnic minorities [4]. Some studies conclude that obesity is more common in blacks [2] while others conclude it is less prevalent [11], and ethnic susceptibility to obesity is not fully documented in the United Kingdom. An understanding of the independent impact of obesity in pregnant women in general, and blacks or ethnic minorities in particular, is important in identifying relevant interventions [12]. Some recent evidence suggested that there might be a substantial difference between ethnic groups in the association of obesity with adverse outcomes [13].

Population attributable fractions (PAFs) are useful in assessing the impact of disease risk factors in populations. They take into account both the strength of the association between a risk factor and an outcome, and the prevalence of the risk factor in the population. There is a limited number of studies looking at PAFs for maternal obesity in the US population [14]–[15] and only one in the Western Europe [16]. The later has examined the PAFs for obesity in a cohort of women living in Netherland on perinatal outcome, the majority of the population being white. No comparable studies have been published in the UK. The importance of PAFs in obstetrics was made poignant in a recent publication which concluded that overweight and obesity may contribute to 40% of stillbirths in developed countries [17].

In this study we aim to assess the impact and contribution of obesity on adverse pregnancy and neonatal outcome in a large ethnically diverse inner-London obstetric population.

## Methods

### Study Design and Setting

A cross sectional analysis of all deliveries at Guy’s and St Thomas’ NHS Foundation Trust between 2004 and 2008 was performed. Data were retrieved from the Guy’s and St Thomas’ NHS Foundation Trust Information System database (Terranova Pacific Services (UK) Ltd, Healthware system) including prospectively collected data between January 1^st^ 2004 and December 31^st^ 2008. This system stores data on all deliveries that take place in the hospital. The data are entered by specially trained midwives and randomly cross-checked by dedicated staff and there have been published work from this data. The software has some prompts, standardised clinical definitions and mandatory fields. All singleton deliveries after 24 weeks of gestation were included in the analysis.

### Variable Definition

#### Exposure variables

Maternal BMI was calculated as weight (kg) at first antenatal visit (booking), divided by height (m) squared. In cases where the data on weight and height were missing from the database, data from the original clinical notes were used. Mothers were classified as: underweight (BMI <18.5), normal (BMI 18.5–24.9), overweight (BMI 25–29.9) and obese (BMI ≥30).

All postcodes obtained from the electronic database were converted into Indices of Multiple Deprivation (IMD) (2007) using the Department of Health and community online converting system [18]. The Index of Multiple Deprivation (IMD) brings together seven different indicators which cover specific aspects or domains of deprivation: Income, Employment, Health and Disability, Education, Skills and Training, Barriers to Housing and Services, Living Environment and Crime. These are weighted and combined to create the overall IMD 2007. Ethnic groups included White (White British, White Irish and Other White), Asian (Bangladeshi, Indian, Pakistani, other Asian and Asian British), Black (Black Caribbean, Black African, other Black and Black British), Mixed (White and Black Caribbean, White and Black African, White and Asian and other mixed), Oriental and Missing (those that had not been recorded).

#### Outcome variables

The primary outcome variables were diabetes in pregnancy (which includes pre-existing diabetes and gestational diabetes) (defined by WHO) [19], Caesarean section (elective and emergency), Instrumental delivery, post-partum haemorrhage status (>500 mls), preterm delivery (delivery less than 37 completed weeks with gestational age assigned by the earliest ultrasound); and for neonatal parameters low birthweight (<2.5 kg), macrosomia (>4 kg), admission to neonatal intensive care unit (NICU) or special care baby unit (SCBU), and perinatal death.

### Statistical Analysis

For obstetric and neonatal outcomes, BMI categories (underweight, normal, overweight and obese) and obesity (yes/no) were used in logistic regression models, with age, deprivation index, ethnicity, parity treated as potential confounders. To test for ethnic variation in the association between obesity and adverse obstetric outcomes, the analyses were stratified by ethnicity and formally tested for interaction by adding an ethnicity-obesity interaction term to the model.

Adjusted population attributable risk fractions (PAFs) for the effect of obesity on different obstetric outcomes were also computed for the whole group, and separately for each ethnic group. The formula used for calculating the PAF was
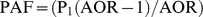
Where:

P_1_ = proportion of women with the condition who are obese.

AOR = Adjusted odds ratio for the association between obesity and the condition of interest.

All analyses were done in Stata software version 11 (Stat Corp, Texas, USA, 2009). The 95% Confidence Intervals for the PAFs are based on the procedure proposed by Greenland and Drescher [25].

The study was approved by Guy’s and St Thomas’ ethics committee and it did not require consent.

## Results

### Sample Description

There were 23,668 singleton deliveries between 2004 and 2008 (an average of 4700 per year), Table 1. Complete data on BMI were available for 17,910 women (76%) and of these 24% were classified as overweight (BMI 25.0–29.9 kg/m^2^) and 14% were classified as obese (BMI equal or greater than 30 kg/m^2^). Fifty five percent of mothers were aged between 25 and 34 years (mean age 31 years), and almost two thirds (62%) were nulliparous. Eighty two percent of the population lived in deprived communities (4^th^ or 5^th^ quintiles IMD) and 46% were from ethnic minority groups: 34% Black, 5% Asian, 3% Oriental and 4% other.

**Table 1:** Background characteristics of the mothers.

Description	Number of women	Percentage
All singleton deliveries	23,668	100%
**Maternal BMI at booking**		
Underweight (<18.5 kg/m^2^)	967	5.40%
Normal weight (18.5–24.9 kg/m^2^)	10,101	56.40%
Overweight (25.0–29.9 kg/m^2^)	4,349	24.30%
Class I obese (30.0–34.9 kg/m^2^)	1,643	9.20%
Class II Obese (35.0–39.9 kg/m^2^)	584	3.30%
Class III or morbidly Obese(≥40.0 kg/m^2^)	266	1.50%
Obese ≥30	2493	14%
Total (non-missing)	17910	100%
* Missing data*	*5758*	*24.30%*
**Maternal age at delivery**		
<20	1,115	4.70%
20–24	3,370	14.20%
25–29	5,528	23.40%
30–34	7,392	31.20%
35–40	4,894	20.70%
>40	1,369	5.80%
Total (non-missing)	23668	100%
* Missing data*	*0*	–
**Parity**		
0	14,753	62.40%
03-Jan	8,384	35.40%
4 plus	528	2.20%
Total (non-missing)	23,665	100%
* Missing data*	*3*	–
**Ethnicity**		
White	12,418	53.80%
Asian or Asian British	1,162	5.00%
Black or Black British	7,793	33.70%
Oriental	736	3.20%
Other	986	4.30%
Total (non-missing)	23,095	100%
* Missing data*	*573*	*2.40%*
**Index of Deprivation**		
1 (least deprived )	638	2.80%
2	1,272	5.60%
3	2,174	9.50%
4	7,091	30.90%
5 (most deprived)	11,776	51.30%
Total (non-missing)	22,951	100%
* Missing data*	*717*	*3%*

The 5,758 women (24%) with missing data on BMI and other variables of interest had similar characteristics to those with BMI data (same mean age of 31, similar proportions of Blacks and Asians and similar distribution of parity: data not shown).

### Association between BMI and Pregnancy Outcome in the Whole Population

Increasing maternal BMI was associated with increasing risk of adverse pregnancy outcome, including diabetes, LSCS and post-partum haemorrhage (table 2). The trend was strongest for diabetes, with odds ratios increasing from 2.38 (95% CI 1.84–3.07) for overweight women, to 9.29 (95% CI 6.64–12.98) for morbidly obese women, compared to women of normal BMI. For emergency LSCS, odds ratios increased from 1.27 (95% CI 1.16–1.39) for overweight women, to 2.05 (95% CI 1.75–2.24) for morbidly obese women, compared to women of normal BMI. Post-partum haemorrhage showed a similar pattern and magnitude of effect. In these examples risks were lowest for underweight women compared to women of normal weight (table 2). A weaker association was seen for preterm delivery, reaching statistical significance in the morbidly obese group (odds ratio 1.77, 95% CI 1.41–2.21).

**Table 2:** Adjusted odds Ratios (95% CI) for obstetric and child outcome according to maternal body mass index.

Characteristic	In wholepopulationN(%)	BMI Category )				
		Underweight<18.5 Kg/m^2^ N(%)aOR^1^ (95%CI)	Normal18.5–24.9Kg/m^2^ N(%)aOR^1^ 95%CI)	Overweight25.0–29.9 Kg/m^2^N(%) aOR^1^(95%CI)	Obese 30.0–34.9Kg/m^2^ N(%) aOR^1^(95%CI)	Morbidly obese>35 Kg/m^2^ N(%)aOR^1^ (95%CI)
**Diabetes**	441 (2.0)	3(0.7) 0.25(0.08–0.77)	131(29.7) 1.0	131(29.7) 2.38(1.84–3.07)	82(18.6) 3.87(2.87–5.22)	94(21.3) 9.29(6.64–12.98)
**Elective LSCS**	1,154 (7.3)	45(3.9) 0.96(0.70–1.33)	562(48.7) 1.0	300(26) 1.21(1.1–1.41)	159(13.8) 1.67(1.38–2.10)	88(7.6) 1.83(1.43–2.35)
**Emergency** **LSCS**	3,948 (22.2)	139(3.5) 0.68(0.56–0.82)	2042(51.7) 1.0	1049(26.6) 1.27(1.16–1.39)	437(11.1) 1.49(1.31–1.69)	288(7.1) 2.05(1.75–2.42)
**Instrumental** **delivery**	2,551 (14.1)	179(7.0) 1.10(0.92–1.32)	1656(64.9) 1.0	514(20.2) 0.82(0.74–0.92)	144(5.6) 0.65(0.54–0.78)	58(2.3) 0.48(0.36–0.64)
**Post-partum** **haemorrhage**	3,722 (20.6)	129(13.4) 0.70(0.57–0.85)	1908(19.0) 1.0	992(23.0) 1.29(1.18–1.41)	414(25.4) 1.47(1.18–1.67)	279(33.0) 2.20(1.88–2.58)
**Preterm** **delivery**	1,346 (8.0)	72(5.4) 1.14(0.88–1.48)	675(50.2) 1.0	343(25.5) 1.12(0.97–1.29)	148(11.0) 1.18(0.97–1.44)	108(8.0) 1.77(1.41–2.21)
**Macrosomia**	1,685 (9.5)	37(2.2) 0.47(0.34–0.67)	832(49.4) 1.0	481(28.6) 1.51(1.33–1.70)	197(11.7) 1.69(1.42–2.01)	138(8.2) 2.37(1.92–2.92)
**Low birth** **weight**	1453(8.1)	109(7.6) 0.64(0.51–0.79)	783(54.6) 1.0	316(22.1) 1.19(1.03–1.37)	136(9.5) 1.13(0.93–1.390)	89(6.2) 0.87(0.68–1.11)
**NICU/SCBU** 2	1,268(6.9)	109(7.6) 0.64(0.51–0.79)	668 (6.6) 1.0	302(6.9) 1.07(0.92–1.23)	149(9.1) 1.42 (1.17–1.72)	90(10.6) 1.67(1.31–2.12)
**Perinatal Death**	112 (0.7)	6(0.6) 1.14 (0.49–2.65)	58(0.6) 1.0	24(0.6) 0.82(0.50–1.33)	17(1.0) 1.38 (0.78–2.45)	7(0.8) 1.07(0.47–2.41)

1 Adjusted for age, parity, deprivation, and ethnic group.

2 NICU/SCBU: Neonatal Intensive Care Unit or Special Care Baby Unit.

For neonatal outcomes, there was a clear association between maternal BMI and macrosomia, odds ratios increasing from 1.5 (95%CI 1.33–1.70) for overweight women, to 2.37 (95% CI 1.92–2.92) for morbidly obese women, compared to women of normal BMI. Increasing maternal BMI was also associated with increasing risk of admission of baby to a neonatal intensive care or special baby unit: odds ratios 1.42(95% CI 01.17–1.72) for obese women and 1.67(95%CI 1.31–2.12) for morbidly obese women, compared to women with normal BMI.

There were few perinatal deaths (112), making the numbers in each BMI category small. Categorising obesity as BMI = >30 Kg/m^2^, obese women were 46% more likely to lose their babies through stillbirth or early neonatal death (OR = 1.46, 95%CI = 0.91–2.32) than women with BMI less than 30 Kg/m^2^(Table 3, first column), but this finding did not reach statistical significance.

**Table 3:** Adjusted odds ratios (95% CI) for obstetric and child outcomes according to maternal obesity^1^, presented for the whole population and separately by ethic group of the mother.

Obstetric and Perinatal outcome	Whole Population aOR 2(95% CI)	Maternal ethnic group				
		WHITE	BLACK	ASIAN	ORIENTAL	
		aOR 2(95% CI)	aOR 2(95% CI)	aOR 2(95% CI)	aOR^2^(95% CI)	P Interaction
**Diabetes**	3.77 (3.08–4.63)	4.97 (3.39–7.28)	2.73 (2.01–3.69)	5.48 (2.43–12.35)	6.62 (1.85–23.67)	0.03
**Elective LSCS**	1.53 (1.31–1.79)	1.41 (1.08–1.84)	1.72 (1.37–2.16)	1.52 (0.73–3.14)	2.16 (0.4–11.59)	0.66
**Emergency LSCS**	1.69 (1.53–1.87)	1.98 (1.69–2.33)	1.39 (1.21–1.60)	0.65 (0.32–1.31)	1.05 (0.34–3.26)	0.60
**Instrumental delivery**	0.64 (0.55–0.74)	0.78 (0.63–0.96)	0.60 (0.45–0.80)	1.04 (0.50–2.16)	1.58 (0.47–5.25)	0.13
**Post-partum haemorrhage**	1.63 (1.47–1.78)	1.75 (1.49–2.06)	1.46 (1.26–1.63)	0.77 (0.40–1.48)	1.38 (0.49–4.05)	0.20
**Preterm Delivery** **(not preterm labour)**	1.43 (1.23–1.65)	1.66 (1.30–2.11)	1.11 (0.91–1.37)	1.25 (0.61–2.56)	1.39 (0.17–11.27)	0.12
**Macrosomia**	1.56 (1.37–1.78)	1.54 (1.27–1.89)	1.68 (1.37–2.07)	0.98 (0.30–3.20)	2.51 (0.65–9.71)	0.10
**Low birth weight**	0.88 (0.76–1.03)	0.75 (0.58–0.98)	1.14 (0.93–1.40)	0.92 (0.47–1.37)	2.18 (0.74–6.38)	0.08
**NICU/SCBU**	1.52 (1.30–1.77)	1.92 (1.52–2.42)	1.22 (0.97–1.52)	1.12 (0.52–2.42)	1.33 (0.16–11.35)	0.05
**Perinatal Death**	1.46 (0.91–2.32)	2.19 (0.96–4.98)	0.80 (0.38–1.67)	2.00 (0.46–8.71)	–	0.11
**Proportion (%) of population obese** 1	14%	9%	24%	9%	3%	

1 BMI 30 or more Kg/m^2^

2 Odds ratios adjusted for maternal age, parity, and deprivation.

### Association between Obesity and Pregnancy Outcome within Ethnic Groups

Table 3 shows the effect of obesity on obstetric outcome within each ethnic group. Obesity was associated with diabetes in all four ethnic groups, and there was evidence of statistical interaction (p<0.03). Odds ratios were highest for the Oriental group (6.62, 95% CI 1.85–23.67) and the Asian group (5.48, 95% CI 2.43–12.35), and lowest for the Black group (2.73, 95% 2.01–3.69).

The effect of maternal obesity on other outcomes showed variability across the ethnic groups, but this variation did not reach statistical significance. There was, however, borderline evidence for interaction between obesity and ethnicity (p = 0.05) in the likelihood of admission of the neonate to a neonatal unit: odds ratios were highest for the White group (1.92, 95% CI 1.52–2.42) and lowest for the Asian group (1.12, 95% CI 0.52–2.42).

### Population Attributable Risk Fractions

Adjusted odds ratios and proportions obese (bottom row of table 3) were used to calculate population attributable risk fraction (PAFs) for obesity in the total population and in each ethnic group. In order of magnitude, PAFs for the total study population were 29% for diabetes, 12% for caesarean section (elective and emergency combined), 7% for macrosomia, 5% for admission to a neonatal unit, and 5% for post-partum haemorrhage. All PAF confidence intervals were positive. The estimate for Perinatal death was 5% but the confidence interval spanned zero (Table 4).

**Table 4:** Population attributable risk fraction (PAF %) for maternal obesity^1^ on obstetric and perinatal outcome.

Obstetric andPerinatal outcome	Whole populationPAF % (95% CI)	Maternal ethnic group			
		WHITE	BLACK	ASIAN	ORIENTAL
		PAF % (95% CI)	PAF % (95% CI)	PAF% (95% CI)	PAF % (95% CI)
Diabetes	29.47 (24.04, 34.70)	20.03 (15.46, 24.53)	41.71 (34.50, 48.1)	17.37 (13.07, 21.09)	8.38 (6.30, 10.42)
Elective CS	6.76 (4.00, 9.44)	4.24 (2.43, 6.00)	11.84 (7.09, 16.34)	4.02 (2.31, 5.70)	1.82 (1.03, 2.59)
Emergency CS	5.18 (3.98, 6.36)	3.48 (2.65, 4.30)	8.19 (6.32, 10.02)	2.93 (2.23, 3.63)	1.38 (1.04, 1.72)
Instrumental delivery	−2.38 (−3.51, −1.27)	−1.84(−2.71, −0.98)	−5.23(−7.73, −2.79)	−1.57(−2.30, −0.84)	−0.70 (−1.02, −0.37)
Post-partum haemorrhage	5.34 (4.06, 6.60)	3.55 (2.67, 4.41)	8.85 (6.76, 10.89)	3.28 (2.47, 4.09)	1.28(0.96, 1.60)
Preterm delivery	4.16 (1.71, 6.55)	2.66 (1.06, 4.23)	6.35 (2.64, 9.91)	2.39 (0.96, 3.81)	1.01 (0.39, 1.63)
Macrosomia	7.38 (5.26, 9.46)	5.15 (3.64, 6.64)	13.20 (9.50, 16.75)	5.52 (3.84, 7.18)	2.08 (1.43, 2.72)
Low birth weight	−0.03 (−0.22, 0.16)	−0.01(−0.10, 0.08)	−0.06(−0.45, 0.33)	−0.03 (−0.20, 0.14)	0.00 (−0.03, 0.02)
NICU-SCBU^2^	5.60 (3.14, 8.01)	3.75 (2.05, 5.41)	9.20 (5.21, 13.02)	3.52 (1.94, 5.07)	1.45 (0.77, 2.12)
Perinatal death	5.20 (−4.71, 14.17)	3.17 (−2.96, 8.93)	7.95 (−7.33, 21.06)	3.02 (−2.78, 8.50)	1.18 (−1.11, 3.41)

1 BMI 30 or more Kg/m^2.^

2 Neonatal Intensive Care Unit or Special Care Baby Unit.

**Figure 1:**
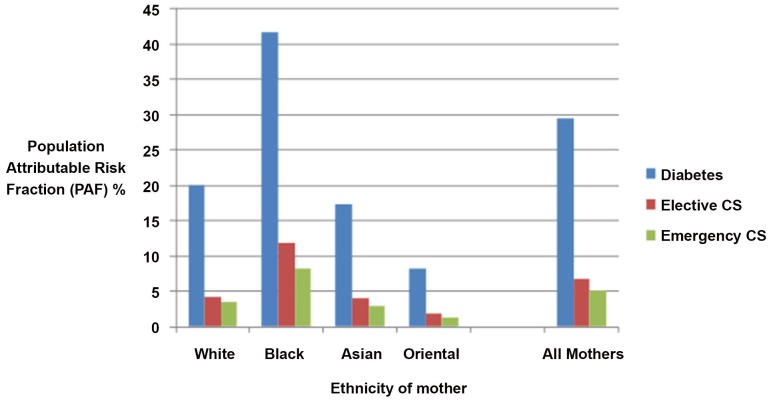
Estimated proportion of diabetes and Caesarean Section which could potentially be avoided if all obese mothers in this population were of normal BMI.

There was a substantial difference in PAFs between different ethnics groups. The contribution of excessive weight to diabetes was the highest in the Black group (42%), followed by that in the White group (20%), the Asian group (17%), and the Oriental group (8%) (figure 1). For elective caesarean section the PAF was again highest in the Black group (12%), 4% for both the White and Asian group, and lowest at 2% for the Oriental group. A similar pattern was seen for Emergency Caesarean section (PAFs in order of magnitude being 8% for Black group, 3% for the White and Asian group, and 1% for the Oriental group, figure 1), postpartum haemorrhage (PAFs in order of magnitude 9% for Black group, 4% for the White, 3% for the Asian group, and 1% for the Oriental group), and preterm delivery (PAFs in order of magnitude 6% for Black group, 3% for the White, 2% for the Asian group, and 1% for the Oriental group).

**Figure 2:**
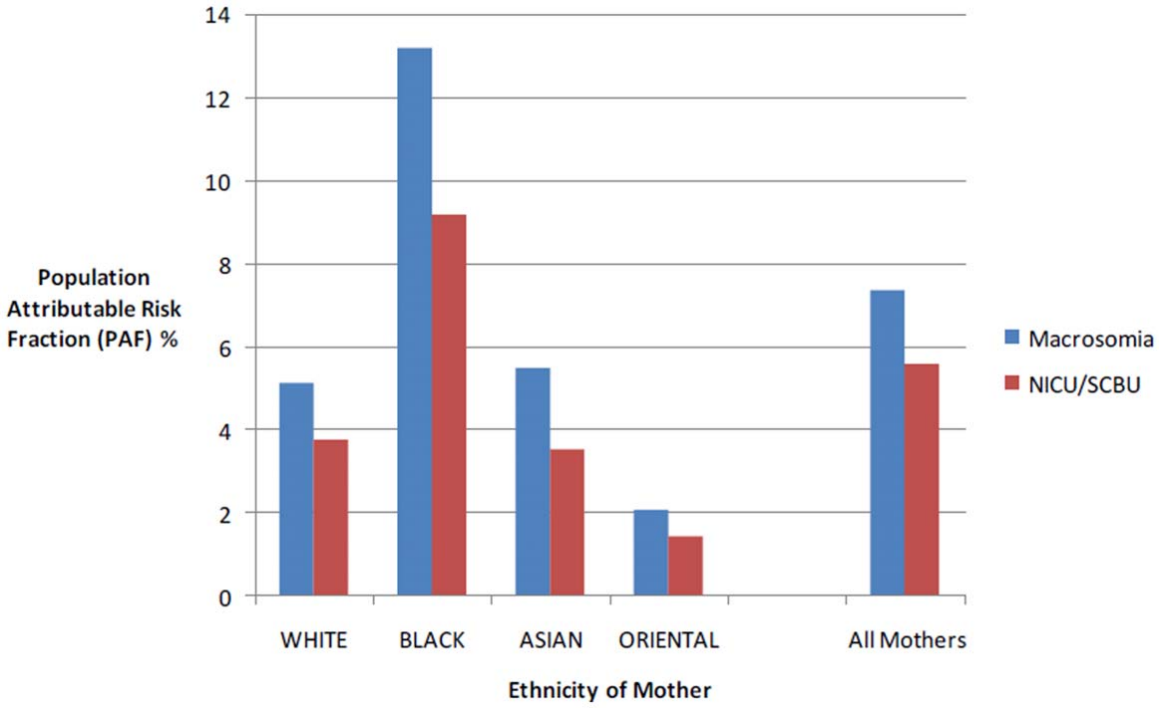
Estimated proportion of Macrosomia and NICU/SCBU admission which could potentially be avoided if all obese mothers in this population were of normal BMI.

For the neonate, PAFs for macrosomia are 13% for the babies of Black mothers, 6% for the babies of Asian mothers, 5% for babies of White mothers and 2% for babies of Orientals mothers (figure 2). For admission to a neonatal care unit PAFs are 9% for the babies of Black mothers, 4% for the babies of White and Asian mothers and 1% for the babies of Oriental mothers (figure 2).

## Discussion

The findings presented here show associations between maternal obesity and adverse obstetric and neonatal events, including diabetes, Caesarean section, preterm birth, post-partum haemorrhage, macrosomia and admission to a neonatal intensive care unit or special care baby unit. This work confirms previous findings on the adverse effects of maternal obesity [4]. Interestingly, the association of obesity with diabetes was strongest for Oriental and Asian women, and lowest for Black women. This observation has been reported only once before in the United Kingdom [13], and concluded that body mass index interacts with racial group with regards to the prevalence of gestational diabetes particularly in South Asian women [13].

A relevant question to ask is how much of the burden of adverse obstetric and neonatal events could be avoided if obesity was eliminated, or at least reduced, in the population? In this study we showed that 29% of diabetes in pregnancy, 12% of Caesarean section, 5% of post-partum haemorrhage, 4% of preterm delivery, 7% of macrosomia, and 5% of admissions to a neonatal intensive care unit or special care baby unit could, potentially, be avoided if there was no maternal obesity in the population. These are, of course, theoretical calculations, but they illustrate the important role obesity plays in determining obstetric morbidity in this population. They also demonstrate the opportunity for substantial cost savings in obstetric health services in this area of South London.

The impact of obesity varied by ethnic group, reflecting differences in the prevalence of obesity. This was most marked for diabetes, where we estimated that 42% of diabetes could be attributed to obesity in the Black population compared to only 8% in the oriental population. In fact, all outcomes examined showed higher population attributable risk fractions for obesity in Black women, driven by the very high prevalence of maternal obesity in this group (24%). Although, at the individual level, obesity had a greater effect in Oriental and Asian women than in Black women, attributable risk fractions were lower because of the lower prevalence of obesity in these groups (3% Orientals and 9% Asian).

The magnitude of the impact of obesity on diabetes (29%) and caesarean section (12%) found in this study was similar to findings reported for the US population of pregnant women [14], which was 30% and 15% respectively. However, the PAF for macrosomia is lower in the current study (7%) than in comparison with others [14], [16] (19% and 15%). This difference could be due to differences in the definition of macrosomia as well as differences in underlying characteristics of the populations.

Obesity is associated with insulin resistance [20]–[21]. Insulin resistance predisposes to diabetes, pre-eclampsia [22], and macrosomia [23]. Macrosomia tends to make vaginal delivery very difficult because of the size of the foetus, and is associated with an increase in Caesarean section rate. Following delivery of macrosomic infant, the uterus is more likely to be atonic and hence predisposed to post partum haemorrhage. Also with a higher Caesarean section rate this also predisposes to post partum haemorrhage. Recent guidelines from RCOG/CMACE and from NICE suggest the importance of managing obesity in pregnancy [11], [24]. This study provides a strong indication that if we are able to reduce obesity in pre-pregnancy, it would have significant impact on maternal and perinatal morbidity and mortality. It also highlights that policies should address the demographic inequality associated with obesity in that it is more common in women from minority ethnic groups, and thus has a greater impact on the black population compared to other ethnic groups.

This paper has highlighted important new findings in obstetrics, but it has limitations. The first is the fact that around one quarter of BMI data were missing. However, we compared the demographic profiles of women with, and without, BMI data and found no difference in terms of mean age, ethnic group, IMD and parity. We do not think the exclusion of women with missing BMI will have biased the study sample. Secondly, we made every effort to address important confounding factors in our analysis but we could not take into account smoking, hypertension, or other obstetric co-morbidity, as this information was not available at the time. However, smoking is known to be strongly associated with socio-economic status, and we were able to control for deprivation but we accept that there may have been a residual confounding at the individual socio-economic level which we will argue has a narrow definition limited only on employment and education of patient and or partner. Thus we think that uncontrolled effects of smoking will have been mitigated to a large degree. Hypertension may be an important confounding factor or effect modifier, or on the causal pathway to adverse outcome, and in future work we will be looking at this in detail in a prospective data collection in order to ensure that hypertension and other co-morbidities are considered. We do not, however, think that obstetric co-morbidity can explain the main findings reported here. Finally, in this study we were unable to distinguish pre-existing diabetes from gestational diabetes so there was some degree of misclassification of outcome. Gestational diabetes accounts for almost 90% of all diabetes in pregnancy. Of the remaining 10%, five percent is type II diabetes (which is also associated with BMI) and the other five percent is type I diabetes [26]. Thus, only a small proportion of diabetes in pregnancy are pre-pregnancy and while we accept that the data is not ideal, we argue that it will be unethical for us to wait for a prospective longitudinal data before publishing our findings.

In conclusion, our study confirms that maternal obesity is linked to maternal and perinatal morbidity for both the individual and the population as a whole. Reducing the prevalence of obesity will reduce the likelihood of adverse events for the obese woman herself and the burden of adverse events in the population. The greatest population impact was seen for diabetes, where 29% of cases could potentially be avoided if all pregnant women were of normal BMI at the start of pregnancy. The impact of obesity is highest for Black women, reflecting the high prevalence of obesity in this group. Policies and strategies to address obesity in pregnancy will have the greatest impact if they target Black women in this population.
